# A Direct Projection from Mouse Primary Visual Cortex to Dorsomedial Striatum

**DOI:** 10.1371/journal.pone.0104501

**Published:** 2014-08-20

**Authors:** Lena A. Khibnik, Nicolas X. Tritsch, Bernardo L. Sabatini

**Affiliations:** Department of Neurobiology, Howard Hughes Medical Institute, Harvard Medical School, Boston, Massachusetts, United States of America; CSIC-Univ Miguel Hernandez, Spain

## Abstract

The mammalian striatum receives inputs from many cortical areas, but the existence of a direct axonal projection from the primary visual cortex (V1) is controversial. In this study we use anterograde and retrograde tracing techniques to demonstrate that V1 directly innervates a topographically defined longitudinal strip of dorsomedial striatum in mice. We find that this projection forms functional excitatory synapses with direct and indirect pathway striatal projection neurons (SPNs) and engages feed-forward inhibition onto these cells. Importantly, stimulation of V1 afferents is sufficient to evoke phasic firing in SPNs. These findings therefore identify a striatal region that is functionally innervated by V1 and suggest that early visual processing may play an important role in striatal-based behaviors.

## Introduction

Understanding the functional circuitry of the basal ganglia requires a thorough characterization of the sources of afferents to the striatum – the principal input structure of the basal ganglia – and their organization within it. Cortex provides one of the major excitatory inputs to the striatum, which was first described by Webster in the 1960s in rat and cat [1], [2]. Similar studies subsequently emerged from other groups describing corticostriatal afferents in rabbit and monkey [3], [4]. These studies indicated that all major cortical areas project to the striatum, noting that inputs from visual cortices targeted rather restricted striatal areas compared to other cortical regions. However, these early studies were performed using focal cortical lesions followed by a silver impregnation of degenerating axons, making it difficult to isolate specific areas of visual cortex and their striatal target regions. Several subsequent investigations have provided conflicting findings: whereas some studies revealed the existence of small corticostriatal inputs from V1 in rodents [5]–[8], others have failed to observe projections originating in V1 in the striatum of monkeys, cats and rodents [9]–[14]. Functional studies have reported visually-driven units in the dorsal striatum of rodents, cats and monkeys [15]–[18], but the relative contributions of primary and extrastriate visual cortices, and of other visual structures such as the superior colliculus are difficult to distinguish *in vivo*. It therefore remains unclear whether V1 functionally innervates the dorsal striatum.

In this study, we examined whether V1 establishes a direct, functional connection with the striatum using a combination of anatomical tracing methods, electrophysiology and optogenetics. We demonstrate that corticostriatal afferents originating in V1 form strong excitatory connections with both direct- and indirect-pathway striatal projection neurons (referred to as dSPNs and iSPNs, respectively), indicating that V1 may directly participate in visually-evoked basal ganglia behaviors.

## Methods

### Mice

All experimental manipulations were performed on mice in accordance with the protocols approved by the Harvard Standing Committee on Animal Care and with the guidelines described in the US National Institutes of Health *Guide for the Care and Use of Laboratory Animals*. All animals used in this study carried the *Rbp4*-Cre transgene (GENSAT #RP24-285K21). For imaging studies, some *Rbp4*-Cre mice were bred to mice bearing an allele encoding EGFP expressed in a Cre-dependent manner under a CAG promoter (referred to as ZSGreen1 reporter transgene; Ai6; the Jackson Laboratory, stock number 007906) to reveal the distribution of Cre^+^ cells. For electrophysiology experiments *Rbp4*-Cre animals were bred to *Drd2-*EGFP transgenic mice (GENSAT #RP23-161H15), which express EGFP under control of a bacterial artificial chromosome containing the type 2 dopamine receptor genomic locus to permit distinction between the direct- and indirect-pathways SPNs. All animals were maintained on a C57Bl/6 background. Both male and female animals were used.

### Viruses and stereotaxic intracranial injections

Conditional expression of EGFP or of the H134R variant of ChR2 in Cre-containing neurons was achieved using recombinant adeno-associated viruses (AAVs) encoding a double-floxed inverted open reading frame (DIO) of ChR2-mCherry or EGFP, as described previously [19], [20]. Briefly, for intracranial injections, mice (postnatal day 30–40) were anesthetized with isoflurane and placed in a small animal stereotaxic frame (David Kopf Instruments). After exposing the skull under aseptic conditions, a small burr hole was drilled for microinjections. All microinjections were performed through a pulled glass pipette using a UMP3 microsyringe pump (World Precision Instruments). Virus (AAV-DIO-EGFP or AAV-DIO-ChR2-mCherry) was injected into V1 (0.3 mm lateral from Lambda, and 0.6 mm below pia) at the rate of 50 nL per minute (500–750 nL total volume). Fluorescent latex microspheres (Red Retrobeads (RRB), Lumafluor) were injected into dorsomedial striatum (0.9 mm anterior and 1.5 mm lateral from Bregma, 2.0 mm below pia) at the rate of 100 nL per minute (150 nL total volume). Following the injection, the skin was sutured using Vicryl 7.0 silk sutures (Ethicon) and mice were returned to their home cage for at least 21 days for AAV injections and 7 days for experiments using fluorescent retrobeads.

### Tissue processing for imaging

Mice were deeply anesthetized with isoflurane and perfused transcardially with 4% paraformaldehyde in 0.1 M sodium phosphate buffer. Brains were post-fixed for 24 hours at room temperature, washed in phosphate buffer saline (PBS) and sectioned (50 µm) coronally using a vibratome (Leica VT1000s). Brain sections were mounted on superfrost slides, dried and coverslipped with ProLong antifade reagent containing DAPI (Molecular Probes). Whole brain sections were imaged with an Olympus VS110 slide-scanning microscope. High-resolution images of regions of interest were subsequently acquired using an Olympus FV1000 confocal microscope (Harvard Neurobiology Imaging Facility). All confocal images were acquired in a single plane and processed using ImageJ.

### Acute slice preparation and electrophysiology

Acute brain slices and whole-cell voltage-clamp recordings from identified SPNs were obtained using standard methods, as described previously [19], [20]. Briefly, mice (50- to 80-days old) were anesthetized by isoflurane inhalation and perfused transcardially with ice-cold artificial cerebrospinal fluid (ACSF) containing (in mM): 125 NaCl, 2.5 KCl, 25 NaHCO_3_, 2 CaCl_2_, 1 MgCl_2_, 1.25 NaH_2_PO_4_ and 11 glucose (290 mOsm per kg). Cerebral hemispheres were removed, placed in cold choline-based cutting solution consisting of (in mM): 110 choline chloride, 25 NaHCO_3_, 2.5 KCl, 7 MgCl_2_, 0.5 CaCl_2_, 1.25 NaH_2_PO_4_, 25 glucose, 11.6 ascorbic acid, and 3.1 pyruvic acid), blocked and transferred into a slicing chamber containing ice-cold choline-based cutting solution. Coronal slices of striatum (275 µm thick) were cut with a Leica VT1000s vibratome, transferred for 10 min to a holding chamber containing ACSF at 34°C and subsequently maintained at room temperature (20–22°C) until use. All recordings were obtained within 4 h of slicing. Both cutting solution and ACSF were constantly bubbled with 95% O_2_/5% CO_2_. Individual slices were transferred to a recording chamber mounted on an upright microscope (Olympus BX51WI) and continuously superfused (2–3 ml per minute) with ACSF at room temperature. Cells were visualized through a 40× water-immersion objective with either infrared differential interference contrast optics or epifluorescence to identify EGFP^+^ iSPNs and striatal regions showing the highest density of ChR2-mCherry^+^ axonal arbors. Epifluorescence was used sparingly to minimize ChR2 activation before recording. Whole-cell voltage- and current-clamp recordings were made from dSPNs and iSPNs in dorsomedial striatum. dSPNs and iSPNs were identified on the basis of the respective absence and presence of EGFP fluorescence and their membrane properties. Patch pipettes (2–4 MΩ) pulled from borosilicate glass (G150F-3, Warner Instruments) were filled either with a Cs^+^-based low Cl^−^ internal solution containing (in mM) 135 CsMeSO_3_, 10 HEPES, 1 EGTA, 3.3 QX-314 (Cl^−^ salt), 4 Mg-ATP, 0.3 Na-GTP, 8 Na_2_-phosphocreatine (pH 7.3 adjusted with CsOH; 295 mOsm per kg) for voltage-clamp recordings, or with a K^+^-based low Cl^−^ internal solution composed of (in mM) 135 KMeSO_3_, 3 KCl, 10 HEPES, 1 EGTA, 3.3 QX-314 (Cl^−^ salt), 4 Mg-ATP, 0.3 Na-GTP, 8 Na_2_-phosphocreatine (pH 7.3 adjusted with KOH; 295 mOsm per kg) for current-clamp recordings. Bath solutions for whole-cell recordings did not contain drugs unless specified otherwise. For all voltage-clamp experiments, errors due to voltage drop across the series resistance (<20 MΩ) were left uncompensated. Membrane potentials were corrected for a ∼8 mV liquid junction potential. To activate ChR2-expressing fibers, light from a 473 nm laser (Optoengine) was focused on the back aperture of the microscope objective to produce wide-field illumination of the recorded cell. Brief pulses of light (1 ms duration; 3–5 mW.mm^−2^ under the objective) were delivered at the recording site at 30 s intervals under control of the acquisition software. Recordings from EGFP^+^ and EGFP^−^ cells were interleaved.

### Data acquisition and analysis

Membrane currents and potentials were amplified and low-pass filtered at 3 kHz using a Multiclamp 700B amplifier (Molecular Devices), digitized at 10 kHz and acquired using National Instruments acquisition boards and a custom version of ScanImage written in MATLAB (Mathworks). Electrophysiology data was analyzed offline using Igor Pro (Wavemetrics) and imaging data was analyzed using ImageJ (National Institutes of Health). Averaged waveforms of 3–5 consecutive sweeps were used to obtain current onset and peak current amplitude. Detection threshold for IPSCs and EPSCs was set at 10 pA. Current onset was measured using a threshold set at three standard deviations of baseline noise. Peak amplitudes were calculated by averaging over a 2 ms window around the peak. For experiments using drug perfusions, peak amplitudes of three consecutive light-evoked responses 3–4 min after drug perfusion onset were averaged, normalized to baseline averages and compared statistically with values obtained at corresponding times in control preparations bathed in ACSF. Data (reported in text and figures as mean ± s.e.m.) were compared statistically using a Mann-Whitney U test. *P* values less than 0.05 were considered statistically significant.

### Reagents

Drugs (all from Tocris) were applied by bath perfusion: gabazine (SR95531; 10 µM), 2,3-dihydroxy-6-nitro-7-sulfamoyl-benzo(*f*)quinoxaline (NBQX; 10 µm), *R*-3-(2-carboxypiperazin-4-yl)propyl-1-phosphonic acid (CPP; 10 µM).

## Results

### Anterograde labeling reveals a direct projection from V1 to dorsomedial striatum (DMS)

**Figure 1:**
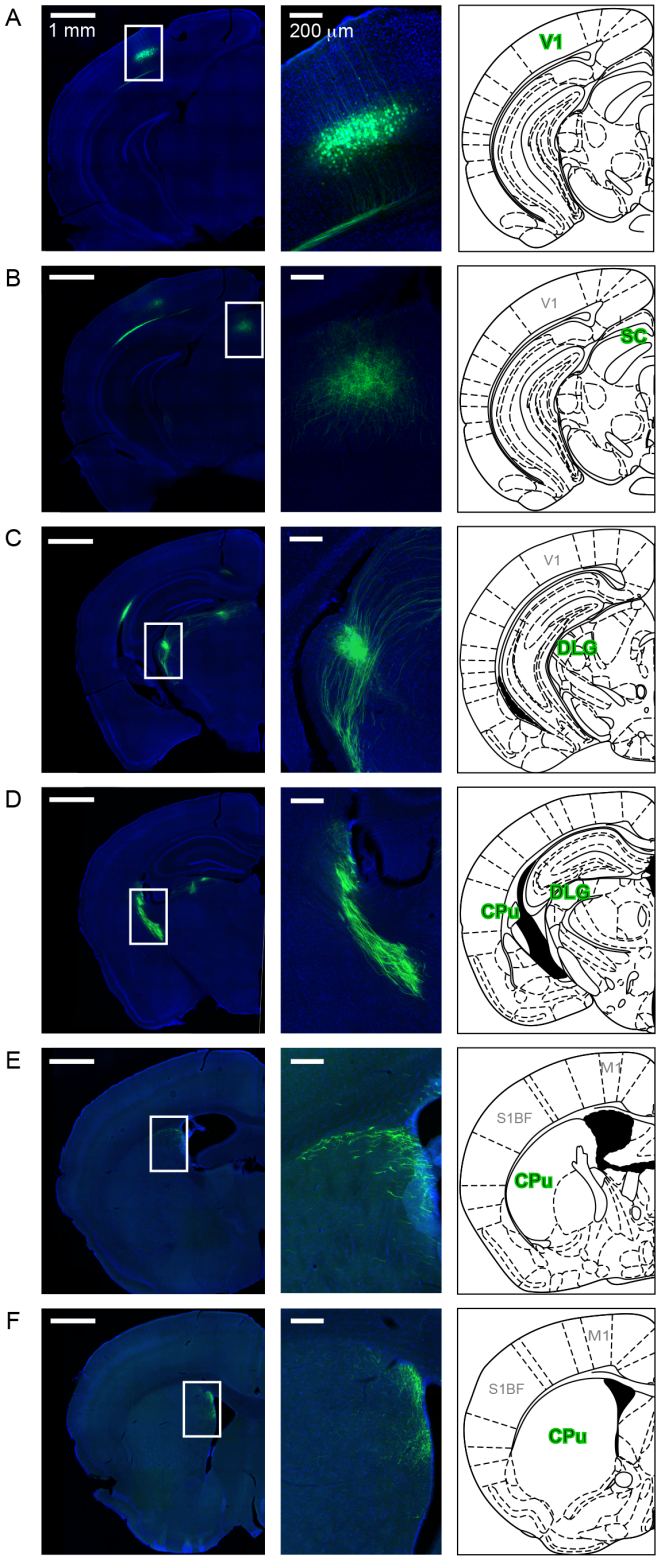
Layer 5 neurons in primary visual cortex project to dorsomedial striatum. **A–F**. Representative serial coronal sections from the brain of a *Rbp4*-Cre mouse virally injected in the primary visual cortex (V1) with an AAV encoding Cre-dependent EGFP to label the cell bodies and axonal processes of neurons in layer 5 (**A**). EGFP-labeled axons leave V1 in through the external capsule (**B**) and innervate various structures including several layers of the superior colliculus (SC; **B**) and dorsal lateral geniculate nucleus (DLG; **C**). V1 axons enter the posterior tail of the striatum (caudate/putamen, CPu; **D**) and course throughout the length of the striatum (**E**) before innervating the anterior dorsomedial quadrant (**F**). *Left*, entire hemisphere stained with DAPI to highlight different brain structures overlaid with the EGFP fluorescence. *Middle*, detailed view of regions outlined in white in the corresponding left panel. *Right*, corresponding images from Paxinos Mouse Brain Atlas highlighting the putative structures where EGFP fluorescence is detected. Similar results were observed eight different mouse brains. M1; primary motor cortex. S1BF; primary somatosensory cortex barrel field.

To address whether layer 5 pyramidal cells in primary visual cortex (V1) project to the striatum of mice, we injected an adeno-associated virus (AAV) encoding Cre-dependent EGFP into V1 of *Rbp4*-Cre transgenic mice, which express Cre in a subset of layer 5 pyramidal neurons throughout cortex [21]. Projecting axon bundles emerging from V1 were readily traced across sections throughout the brain of 8 mice, and followed stereotyped trajectories and innervation patterns in all brains studied (Figure 1A–F). As axons exited V1, EGFP-labeled axons formed a thick bundle that traveled ventrally in the ipsilateral external capsule (Figure 1A–B). These axons innervated well-defined projection areas, including the superior colliculus (Figure 1B) and the dorsal part of the lateral geniculate nucleus (Figure 1C–D), confirming the site of injection as V1 [22]–[24]. Interestingly, V1 axons also left the external capsule (Figure 1D) to enter the most posterior part of the ipsilateral striatum and then coursed anteriorly within the striatum in tight axonal bundles lining dorsal and medial regions. Terminal fields, which were identified by the splaying of fibrils throughout the neuropil, were observed most prominently in the anterior half of the DMS (Figure 1E–F). Corticostriatal axons from V1 targeted a restricted longitudinal strip of the ipsilateral DMS lining the lateral ventricle, forming two dense innervation regions: one at the more dorsal aspect of the strip and the other on its ventral side. At higher magnification, sparse axons were occasionally detected in more medial parts of dorsal striatum (data not shown). Interestingly, V1 neurons also extended axons medially and anteriorly towards the corpus callosum to innervate the contralateral V1 as well as the contralateral DMS (not shown). These axonal projections were comparatively much more sparse and weakly labeled, and were consequently not studied further. Our results therefore reveal that layer 5 pyramidal neurons in the mouse primary visual cortex extend bilateral axonal projections into the striatum.

### Retrograde labeling of V1 projections to DMS

**Figure 2:**
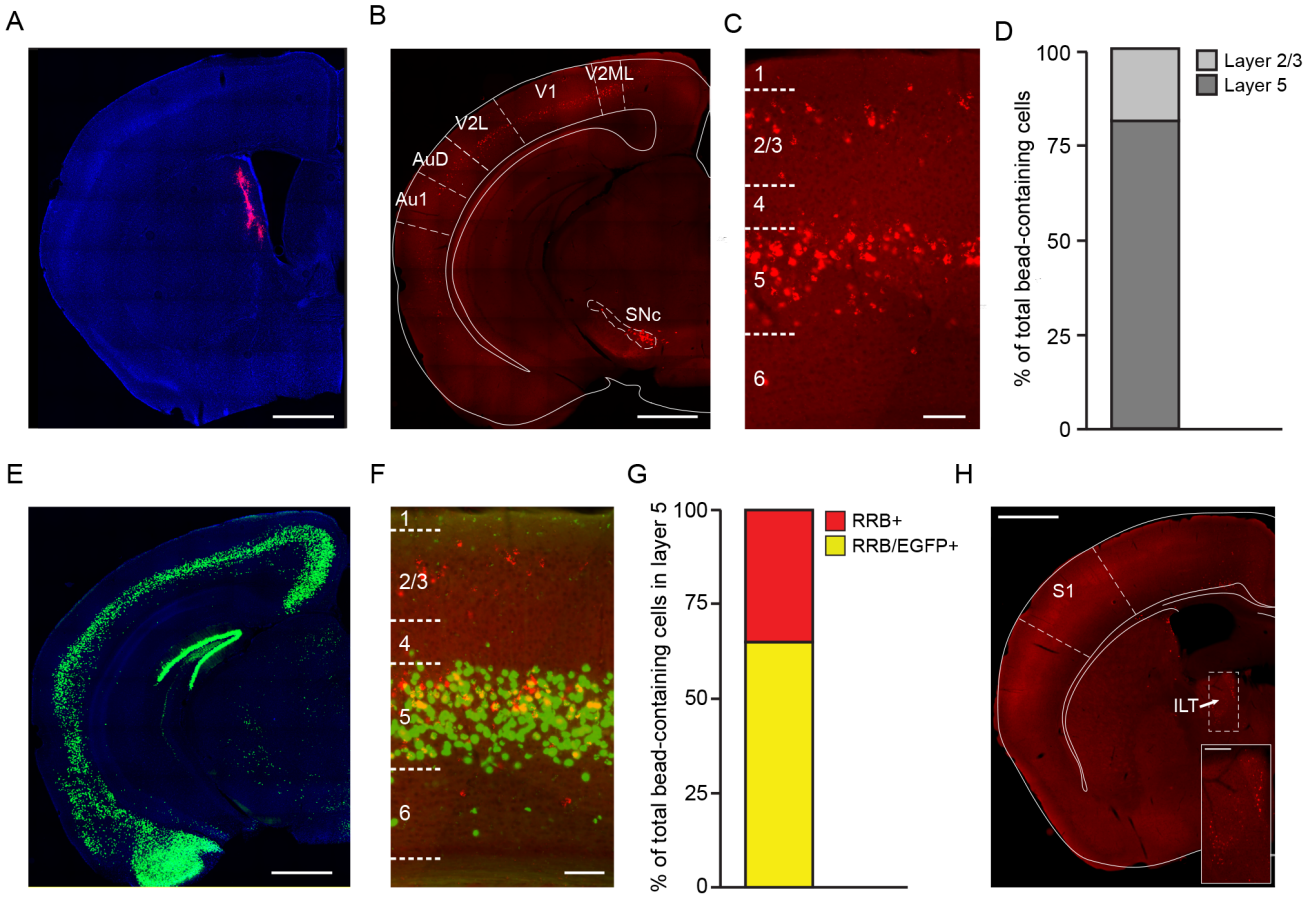
Retrograde labeling of V1 neurons innervating the DMS. **A**. Coronal striatal section showing the site of red retrobead (RRB) injection into the dorsomedial striatum (DMS). The section is counterstained with DAPI. **B**. Coronal brain section of V1 containing RRBs retrogradedly transported from the dorsomedial striatum. Secondary visual cortex (V2ML and V2L) and secondary auditory cortex (AuD), but not Au1 (primary auditory cortex) were also labeled. As expected, retrobeads were also detected in putative dopaminergic neurons within the substantia nigra pars compacta (SNc). **C**. Detailed view of V1 showing the relative distribution of retrobead-labeled cells bodies across cortical layers. **D**. Distribution of retrobead-labeled neurons in V1 across cortical layers. The majority (81%) of labeled cells are situated within layer 5, while only 19% distribute to layers 2/3. **E**. Representative coronal section from a *Rbp4*-Cre;ZsGreen1 brain showing EGFP fluorescence concentrated in the cell bodies of Cre-containing cortical neurons in layer 5. The section is counterstained with DAPI. **F**. Detailed view of V1 in a *Rbp4*-Cre;ZsGreen1 brain injected with RRBs in the DMS illustrating the extensive overlap between retrobead- and Cre-containing (EGFP-positive) cells in upper layer 5. **G**. Percentage of all retrobead-labeled layer 5 neurons that also contain Cre (RRB/EGFP^+^; 65%, shown in yellow) vs. cells that are only RRB^+^ (35%, red). **H**. Coronal brain section showing red retrobead labeled cells in thalamus. ILT: Intralaminar thalamic nuclei. Scale: 1 mm for panels A, B, E and H; 100 µm for panels C and F.

To determine whether axons originating in V1 establish synaptic contacts within the DMS, we employed a retrograde labeling method using fluorescently labeled microspheres (referred to as red retrobeads or RRBs) that are absorbed by the terminal zones of axons and transported back to the cell body [25]. Retrobeads are not transported trans-synaptically and therefore only label the soma of neurons that directly project into the injection region. Following stereotaxic injection of RRBs in the DMS (Figure 2A), we observed retrogradely labeled cells in several brain regions known to innervate dorsal striatum, including the substantia nigra pars compacta (SNc; Figure 2B) as well as the intralaminar thalamus, a region providing the densest thalamic input to the striatum [23] (Figure 2H). Importantly, we observed dense retrograde labeling within V1, confirming that V1 innervates DMS. Interestingly, microsphere-labeled cells were not found in either auditory (Figure 2B) or somatosensory cortex (Figure 2H), indicating that these cortical areas do not extend sizeable axonal arbors into the medial-most region of dorsal striatum. These observations are consistent with the notion that cortical inputs into the dorsal striatum are largely topographic, with primary auditory cortex innervating primarily the posterior portion of striatum and primary somatosensory cortex projections occupying mainly the dorsolateral aspect of anterior striatum ([6], [14], Allen Institute for Brain Science Mouse Brain Connectivity Atlas (http://connectivity.brain-map.org) [26]).

The majority of pyramidal neurons in somatosensory and motor cortices that project to the striatum lie within the upper half of layer 5, although corticostriatal neurons in layer 2/3 have also been described [6], [27], [28]. Consistent with the distribution of corticostriatal neurons in these other brain regions, most of the retrogradely labeled cell bodies in V1 rested within layer 5 (Figure 2C): out of 1159 retrogradely-labeled cortical cells in V1 (n = 4 mice), 943 (81%) distributed to layer 5, while only 216 (19%) were in layers 2/3 (Figure 2D). On rare occasions, cells in layer 6 were observed, but they constituted a negligible percentage of the total number of corticostriatal neurons.

Because the transgenic line used in our anterograde study (*Rbp4*-Cre; Figure 1) restricts Cre expression to layer 5 neocortical neurons in V1, it represents an attractive tool for studying the function of visual cortex neurons projecting to striatum. To determine the fraction of corticostriatal neurons expressing Cre in these mice, we injected red fluorescent retrobeads in the DMS of *Rbp4*-Cre mice expressing EGFP in the soma of Cre-containing neurons (ZSGreen1, Figure 2E). Out of 531 layer 5 cells in V1 that innervate striatum (RRB-positive), the majority (345, 65%) also expressed Cre (EGFP^+^) and the remaining 186 cells (35%) were EGFP^−^ (Figure 2G). This indicates that the *Rbp4*-Cre line expresses Cre in two-thirds of layer 5 corticostriatal neurons, confirming the usefulness of this line in labeling striatally-projecting cortical neurons. Interestingly, the majority of neurons double-labeled for EGFP and RBB distributed to upper layer 5, suggesting that most of the DMS-projecting corticostriatal neurons labeled in *Rbp4*-Cre mice are intratelencephalic (IT) cortical cells [29]–[32].

### V1 inputs into DMS drive action potentials in SPNs

**Figure 3:**
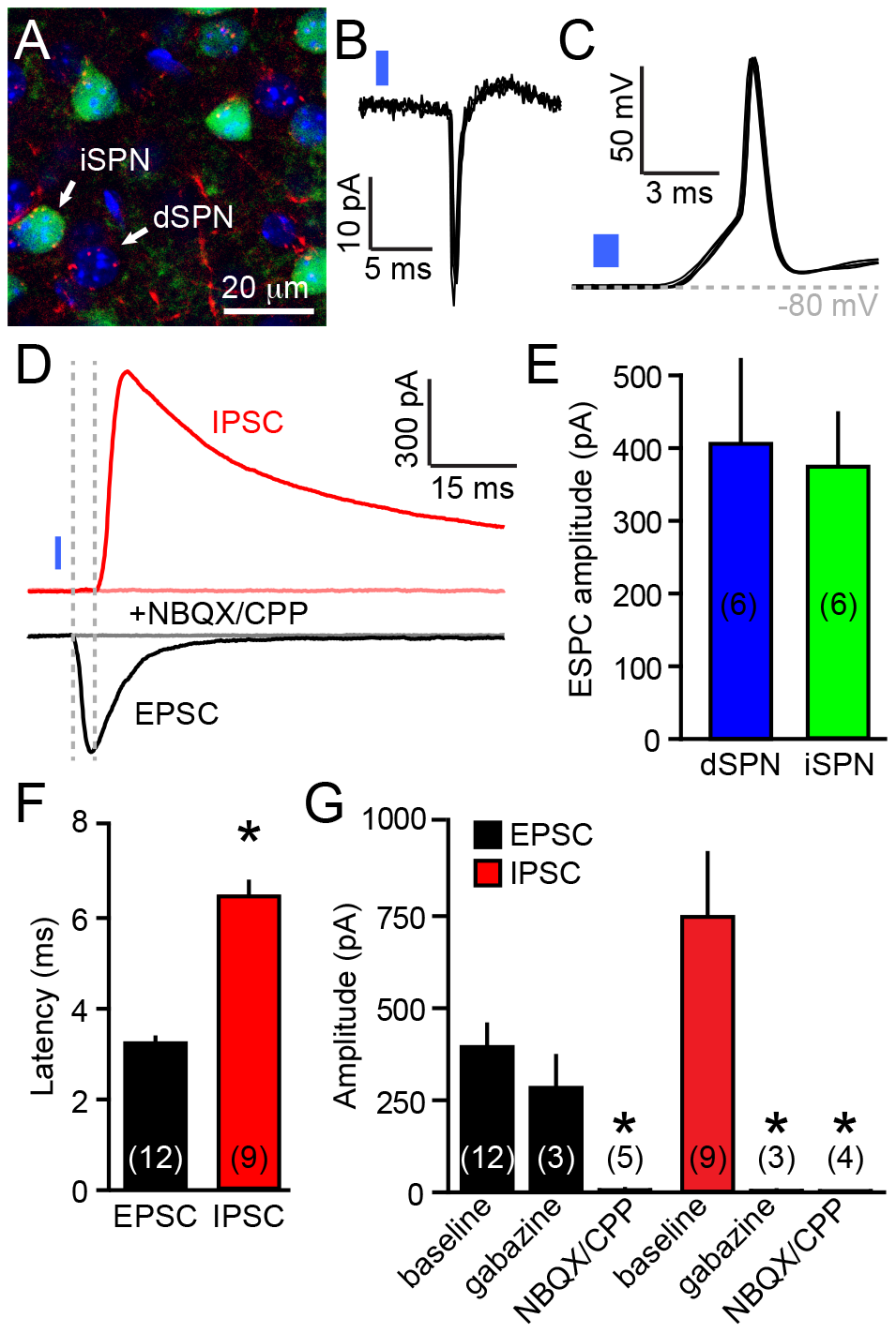
Stimulation of V1 axons engages glutamatergic and GABAergic synaptic transmission onto SPNs. **A**. Representative high magnification view of the dorsomedial striatum in a *Rbp4*-Cre; *Drd2-*EGFP mouse virally injected in V1 with an AAV encoding Cre-dependent ChR2-mCherry. Axonal projections from V1 (red) are clearly seen within the DMS. The section is stained with DAPI (blue) to label cell bodies. iSPNs are easily identified using EGFP fluorescence (green). A presumptive dSPN (DAPI^+^; EGFP^−^) is also shown. **B**. Cell-attached recording from a SPN showing that a 1 ms 473 nm light flash (blue bar) reliably evokes a single action potential. Three consecutive extracellular waveforms are overlaid. **C**. As in **B** for another SPN recorded in the whole-cell current-clamp configuration. **D**. Whole-cell voltage-clamp traces from a SPN upon optogenetic stimulation (1 ms, blue bar) of ChR2-expressing V1 axons. EPSCs (black) were recorded at *E*_Cl_ = −70 mV, while IPSCs (red) were recorded at 0 mV (the reversal potential for ionotropic glutamate receptor mediated currents). Dashed gray lines mark the onset of both currents and highlight the delayed onset of IPSCs relative to EPSCs. Both EPSC and IPSC were eliminated after bath application of the glutamate receptor antagonists NBQX and CPP (both at 10 µM; gray and pink lines, respectively), confirming the disynaptic origin of IPSCs. **E**. Mean (± s.e.m) peak EPSC amplitude in dSPNs (blue) and iSPNs (green). **F**. Mean (± s.e.m) latency from flash onset to current onset of EPSCs (black) and IPSCs (red). **G**. Mean (± s.e.m) EPSC (black) and IPSC (red) amplitudes. Asterisk represents statistical significance, *P*<0.05 vs. baseline amplitude. Data in E–G represent mean ± s.e.m. Number of recordings are indicated in parentheses.

To determine whether V1 corticostriatal neurons form functional synaptic connections in the striatum, we expressed channelrhodopsin (ChR2-mCherry) in V1 of *Rbp4*-Cre;*Drd2-*EGFP mice and obtained recordings from direct- (dSPN) and indirect-pathway striatal projection neurons (iSPNs) in coronal sections of DMS (Figure 3A). Stimulation of V1 axons using 1 ms light flashes reliably evoked action potentials in SPNs in both cell-attached (n = 4; Figure 3B) and whole-cell current-clamp recording configurations (n = 3; Figure 3C), indicating that corticostriatal axons originating in V1 are functional and strong enough to reliably depolarize SPNs above spike threshold. We used whole-cell voltage-clamp recordings from SPNs to characterize the synaptic conductances evoked by optogenetic activation of V1 axons. In SPNs clamped at −70 mV (the Cl^−^ reversal potential), ChR2 stimulation reliably evoked large excitatory postsynaptic currents (EPSCs) in both dSPNs and iSPNs (Figures 3D–E) without significant differences in kinetics (not shown) or mean amplitude of EPSCs across pathways (EGFP^+^ iSPN: −376±73 pA, n = 6; EGFP^−^ dSPN: −406±119 pA, n = 6; *P* = 0.9, Mann-Whitney U test; Figure 3E). Data from both neuronal populations were consequently combined for further analyses. EPSCs in SPNs displayed short synaptic latencies at ambient temperature (3.2±0.2 ms, n = 12; Figure 3F) and were eliminated by a cocktail of α-amino-3-hydroxy-5-methyl-4-isoxazolepropionic acid (AMPA) and *N*-methyl-D-aspartate (NMDA) receptor antagonists (mean amplitude: −6±4 pA, n = 5; *P*<0.01 vs. baseline, Mann-Whitney U Test; Figure 3D, G), indicating that they result from monosynaptic activation of ionotropic glutamate receptors. As anticipated, the GABA_A_ receptor antagonist gabazine did not affect EPSCs (mean amplitude: −283±92 pA, n = 3; *P* = 0.5 vs. baseline; Mann-Whitney U Test; Figure 3G).

Stimulation of corticostriatal afferents in slice potently engages feed-forward inhibition, which is mediated by striatal fast spiking interneurons and SPN collaterals [33]. To determine if corticostriatal afferents arising in V1 similarly recruit local inhibitory circuits in striatum, we recorded light-evoked responses in SPNs clamped at 0 mV (the reversal potential of ionotropic glutamate receptors) to reveal inhibitory postsynaptic currents (IPSCs). Under these conditions, we consistently observed large IPSCs (mean amplitude: 746±182 pA, n = 9) that were blocked by gabazine (mean amplitude: 4±2 pA, n = 3; *P*<0.01 vs. baseline, Mann-Whitney U Test), indicating that corticostriatal stimulation triggers synaptic release of GABA (Figures 3D,G). Consistent with a disynaptic origin, IPSCs displayed synaptic latencies twice as long as those of EPSCs (6.3±0.4 ms, n = 9; P<0.001, Mann Whitney U Test; Figure 3F) and were eliminated in the presence of NBQX and CPP (mean amplitude: 3±1 pA, n = 4; *P*<0.01 vs. baseline, Mann-Whitney U Test; Figure 3D,G).

## Discussion

The dorsal striatum plays a crucial role in motor planning, motivation, as well as procedural and reward learning behaviors [34]–[37]. As one of the major sources of excitatory inputs into the dorsal striatum, cortex provides an important signal that shapes striatal function and subsequent behavioral outcomes [38], [39]. Our results reveal that, in addition to well-established somatosensory, auditory and motor corticostriatal afferents, the striatum of mice also receives strong synaptic inputs from primary visual cortex in its dorsal-medial quadrant. This finding therefore suggests that early visual processing may play an important role in visually guided striatal-based behaviors.

Studies dating back to the 1960s have attempted to map corticostriatal inputs [1]–[4], [6], [12]. Many of these and subsequent studies investigated whether V1 provides a direct synaptic input into the striatum, but the existence of this connection has remained controversial. Several experimental factors might have contributed to conflicting reports. First, these studies were performed across a wide variety of species, including monkeys, rats, rabbits, and hamsters. Second, some labeling methods might have lacked the sensitivity required to reveal this projection. Third, the use of focal cortical lesions for mapping corticostriatal inputs might have presented difficulties in isolating V1 from neighboring regions. In this study, we employed sensitive labeling, imaging and optogenetic methods to conclusively demonstrate that V1 establishes a direct and functionally strong axonal projection into the DMS in mice. Using viral expression of EGFP in V1 and injection of retrogradely transported beads in the DMS, we confirmed that V1 neurons innervate a longitudinal strip of the DMS that lines the ventricle. In agreement with the distribution of corticostriatal afferents in other cortical regions, we found that V1 neurons projecting to the DMS lie in both layer 5 and layers 2/3, with the overwhelming majority of them in upper layer 5. Moreover, we used optogenetics and electrophysiological recordings from SPNs to show that the axons of neocortical L5 neurons in V1 functionally innervate striatum. Our anatomical findings are supported by studies in rats [5], [6], [14], as well as in mice (Allen Institute for Brain Science Mouse Brain Connectivity Atlas, [26]), which reported a similar projection from V1 that mainly terminates ipsilaterally in a longitudinal strip of the DMS.

Two broad groups of corticostriatal afferents can be distinguished anatomically: intra-telencephalic (IT) neurons mainly distribute to upper layer 5 and innervate the striatum bilaterally, while pyramidal tract (PT) neurons rest in lower layer 5 and extend collaterals ipsilaterally into the striatum, thalamus and midbrain on their way to the brainstem and spinal cord [29]–[32]. Although we did not observe V1 axons in the ponds or pyramidal tract, EGFP^+^ axons were clearly seen in contralateral striatum and ipsilateral thalamus and superior colliculus, indicating that the layer 5 pyramidal neurons labeled in the primary visual cortex of *Rbp4*-Cre mice comprise both IT and PT-type cortical cells, in agreement with previous reports [40]. Several lines of evidence suggest that the layer 5 neurons in V1 that innervate the DMS likely belong to the IT group. First, V1 neurons innervate the DMS bilaterally. Second, most of the Cre^+^ neurons identified with a retrograde signal from the DMS (retrobeads) are situated in upper layer 5 (Figure 2F). Third, our electrophysiological data did not reveal preferential targeting of dSPNs vs. iSPNs by V1 afferents, in agreement with recent anatomical and functional studies in mice that did not report significant differences in the relative innervation of dSPNs and iSPNs by IT-type corticostriatal neurons in sensory cortices [41], [42]. These data therefore indicate that although *Rbp4*-Cre mice express Cre in both IT and PT neurons, the visual cortex neurons that innervate the DMS are mostly of the IT-type.

Our study also confirms the usefulness of the *Rbp4*-Cre line for the study of corticostriatal afferents. We found that it expresses Cre in the majority of layer 5 neurons that project to the striatum. Although the fluorescent reporter line used in this study might not capture all Cre-expressing cells, the roughly one third of unlabeled corticostriatal neurons in layer 5 likely represent a population of cells in which Cre is not expressed. Study of these corticostriatal afferents as well as those originating in layer 2/3 will therefore require another Cre driver line. Importantly, Cre expression is not strictly limited to corticostriatal neurons as neurons in the entorhinal cortex and hippocampus also contain Cre (Figure 2E), and layer 5 neurons in V1 also extended axonal projections into the superior colliculus and thalamus (Figure 1). Although it remains to be determined whether these projections constitute collaterals of corticostriatal neurons or whether they arise from a distinct population of layer 5 neurons, this observation indicates that care should be taken when interpreting the effects of opto- and pharmacogenetic manipulations of these neurons on behavior. Nevertheless, the *Rbp4*-Cre transgenic line should prove useful in studying general principles of corticostriatal transmission and circuitry.

Together, our findings indicate that corticostriatal inputs originating in primary visual cortex can efficiently recruit neuronal circuits in defined regions of the striatum, suggesting that early visual processing might contribute to corticostriatal synaptic plasticity as well as visually guided motor behaviors.

## References

[1] Webster KE (1961) Cortico-striate interrelations in the albino rat. J Anat 95: 532–544.3. 14005491 PMC1244066

[2] Webster KE (1965) The cortico-striatal projection in the cat. J Anat 99: 329–337. 14327177 PMC1261395

[3] Carman JB, Cowan WM, Powell TP (1963) The Organization of Cortico-Striate Connexions in the Rabbit. Brain 86: 525–562. 14063897 10.1093/brain/86.3.525

[4] Kemp JM, Powell TP (1970) The cortico-striate projection in the monkey. Brain 93: 525–546. 4990231 10.1093/brain/93.3.525

[5] Faull RL, Nauta WJ, Domesick VB (1986) The visual cortico-striato-nigral pathway in the rat. Neuroscience 19: 1119–1132. 3822114 10.1016/0306-4522(86)90128-4

[6] McGeorge AJ, Faull RL (1989) The organization of the projection from the cerebral cortex to the striatum in the rat. Neuroscience 29: 503–537. 2472578 10.1016/0306-4522(89)90128-0

[7] Rhoades RW, Kuo DC, Polcer JD, Fish SE, Voneida TJ (1982) Indirect visual cortical input to the deep layers of the hamster's superior colliculus via the basal ganglia. J Comp Neurol 208: 239–254. 7119160 10.1002/cne.902080304

[8] Rhoades RW, Mooney RD, Fish SE (1985) Subcortical projections of area 17 in the anophthalmic mouse. Brain Res 349: 171–181. 3986586 10.1016/0165-3806(85)90141-5

[9] Battaglini PP, Squatrito S, Galletti C, Maioli MG, Sanseverino Riva E (1982) Bilateral projections from the visual cortex to the striatum in the cat. Exp Brain Res 47: 28–32. 7117439 10.1007/BF00235882

[10] Saint-Cyr JA, Ungerleider LG, Desimone R (1990) Organization of visual cortical inputs to the striatum and subsequent outputs to the pallido-nigral complex in the monkey. J Comp Neurol 298: 129–156. 1698830 10.1002/cne.902980202

[11] Updyke BV (1993) Organization of visual corticostriatal projections in the cat, with observations on visual projections to claustrum and amygdala. J Comp Neurol 327: 159–193. 8381142 10.1002/cne.903270202

[12] Pan WX, Mao T, Dudman JT (2010) Inputs to the dorsal striatum of the mouse reflect the parallel circuit architecture of the forebrain. Front Neuroanat 4: 147. 21212837 10.3389/fnana.2010.00147 PMC3014656

[13] Veening JG, Cornelissen FM, Lieven PA (1980) The topical organization of the afferents to the caudatoputamen of the rat. A horseradish peroxidase study. Neuroscience 5: 1253–1268. 7402468 10.1016/0306-4522(80)90198-0

[14] López-Figueroa MO, Ramirez-Gonzalez JA, Divac I (1995) Projections from the visual areas to the neostriatum in rats. A re-examination. Acta Neurobiol Exp (Wars) 55: 165–175. 8553910 10.55782/ane-1995-1073

[15] Hikosaka O, Sakamoto M, Usui S (1989) Functional properties of monkey caudate neurons. II. Visual and auditory responses. J Neurophysiol 61: 799–813. 2723721 10.1152/jn.1989.61.4.799

[16] Strecker RE, Steinfels GF, Abercrombie ED, Jacobs BL (1985) Caudate unit activity in freely moving cats: effects of phasic auditory and visual stimuli. Brain Res 329: 350–353. 3978457 10.1016/0006-8993(85)90548-7

[17] Schulz JM, Redgrave P, Reynolds JNJ (2010) Cortico-striatal spike-timing dependent plasticity after activation of subcortical pathways. Front Synaptic Neurosci 2: 23. 21423509 10.3389/fnsyn.2010.00023 PMC3059678

[18] Nagy A, Eördegh G, Norita M, Benedek G (2003) Visual receptive field properties of neurons in the caudate nucleus. Eur J Neurosci 18: 449–452. 12887427 10.1046/j.1460-9568.2003.02764.x

[19] Tritsch NX, Ding JB, Sabatini BL (2012) Dopaminergic neurons inhibit striatal output through non-canonical release of GABA. Nature 490: 262–266. 23034651 10.1038/nature11466 PMC3944587

[20] Saunders A, Johnson CA, Sabatini BL (2012) Novel recombinant adeno-associated viruses for Cre activated and inactivated transgene expression in neurons. Front Neural Circuits 6: 47. 22866029 10.3389/fncir.2012.00047 PMC3406316

[21] Gong S, Doughty M, Harbaugh CR, Cummins A, Hatten ME, et al (2007) Targeting Cre recombinase to specific neuron populations with bacterial artificial chromosome constructs. J Neurosci 27: 9817–9823. 17855595 10.1523/JNEUROSCI.2707-07.2007 PMC6672645

[22] Dräger UC, Hubel DH (1976) Topography of visual and somatosensory projections to mouse superior colliculus. J Neurophysiol 39: 91–101. 1249606 10.1152/jn.1976.39.1.91

[23] Smith Y, Raju D, Nanda B, Pare J-F, Galvan A, et al (2009) The thalamostriatal systems: anatomical and functional organization in normal and parkinsonian states. Brain Res Bull 78: 60–68. 18805468 10.1016/j.brainresbull.2008.08.015 PMC2656644

[24] Sillito AM, Jones HE (2002) Corticothalamic interactions in the transfer of visual information. Philos Trans R Soc Lond B Biol Sci 357: 1739–1752. 12626008 10.1098/rstb.2002.1170 PMC1693075

[25] Katz LC, Burkhalter A, Dreyer WJ (1984) Fluorescent latex microspheres as a retrograde neuronal marker for in vivo and in vitro studies of visual cortex. Nature 310: 498–500. 6205278 10.1038/310498a0

[26] Oh SW, Harris JA, Ng L, Winslow B, Cain N, et al (2014) A mesoscale connectome of the mouse brain. Nature 508: 207–214. 24695228 10.1038/nature13186 PMC5102064

[27] Gerfen CR (1989) The neostriatal mosaic: striatal patch-matrix organization is related to cortical lamination. Science 246: 385–388. 2799392 10.1126/science.2799392

[28] Rosell A, Giménez-Amaya JM (1999) Anatomical re-evaluation of the corticostriatal projections to the caudate nucleus: a retrograde labeling study in the cat. Neurosci Res 34: 257–269. 10576548 10.1016/s0168-0102(99)00060-7

[29] Reiner A, Jiao Y, Del Mar N, Laverghetta AV, Lei WL (2003) Differential morphology of pyramidal tract-type and intratelencephalically projecting-type corticostriatal neurons and their intrastriatal terminals in rats. J Comp Neurol 457: 420–440. 12561080 10.1002/cne.10541

[30] Lei W, Jiao Y, Del Mar N, Reiner A (2004) Evidence for differential cortical input to direct pathway versus indirect pathway striatal projection neurons in rats. J Neurosci 24: 8289–8299. 15385612 10.1523/JNEUROSCI.1990-04.2004 PMC6729697

[31] Wilson CJ (1987) Morphology and synaptic connections of crossed corticostriatal neurons in the rat. J Comp Neurol 263: 567–580. 2822779 10.1002/cne.902630408

[32] Anderson CT, Sheets PL, Kiritani T, Shepherd GMG (2010) Sublayer-specific microcircuits of corticospinal and corticostriatal neurons in motor cortex. Nat Neurosci 13: 739–744. 20436481 10.1038/nn.2538 PMC2876193

[33] Tepper JM, Wilson CJ, Koós T (2008) Feedforward and feedback inhibition in neostriatal GABAergic spiny neurons. Brain Res Rev 58: 272–281. 18054796 10.1016/j.brainresrev.2007.10.008 PMC2562631

[34] Yin HH, Knowlton BJ (2006) The role of the basal ganglia in habit formation. Nat Rev Neurosci 7: 464–476. 16715055 10.1038/nrn1919

[35] Graybiel AM, Aosaki T, Flaherty AW, Kimura M (1994) The basal ganglia and adaptive motor control. Science 265: 1826–1831. 8091209 10.1126/science.8091209

[36] Packard MG, Knowlton BJ (2002) Learning and memory functions of the Basal Ganglia. Annu Rev Neurosci 25: 563–593. 12052921 10.1146/annurev.neuro.25.112701.142937

[37] Hikosaka O, Takikawa Y, Kawagoe R (2000) Role of the basal ganglia in the control of purposive saccadic eye movements. Physiol Rev 80: 953–978. 10893428 10.1152/physrev.2000.80.3.953

[38] Koralek AC, Jin X, Long JDII, Costa RM, Carmena JM (2012) Corticostriatal plasticity is necessary for learning intentional neuroprosthetic skills. Nature 483: 331–335. 22388818 10.1038/nature10845 PMC3477868

[39] Znamenskiy P, Zador AM (2013) Corticostriatal neurons in auditory cortex drive decisions during auditory discrimination. Nature 497: 482–485. 23636333 10.1038/nature12077 PMC3670751

[40] Gerfen CR, Paletzki R, Heintz N (2013) GENSAT BAC cre-recombinase driver lines to study the functional organization of cerebral cortical and basal ganglia circuits. Neuron 80: 1368–1383. 24360541 10.1016/j.neuron.2013.10.016 PMC3872013

[41] Kress GJ, Yamawaki N, Wokosin DL, Wickersham IR, Shepherd GMG, et al (2013) Convergent cortical innervation of striatal projection neurons. Nat Neurosci 16: 665–667. 23666180 10.1038/nn.3397 PMC4085670

[42] Wall NR, De La Parra M, Callaway EM, Kreitzer AC (2013) Differential innervation of direct- and indirect-pathway striatal projection neurons. Neuron 79: 347–360. 23810541 10.1016/j.neuron.2013.05.014 PMC3729794

